# Correction to: decreasing hospital burden of COVID-19 during the first wave in Regione Lombardia: an emergency measures context

**DOI:** 10.1186/s12889-021-12144-2

**Published:** 2021-11-15

**Authors:** Francesca Maria Grosso, Anne Margaret Presanis, Kevin Kunzmann, Chris Jackson, Alice Corbella, Giacomo Grasselli, Aida Andreassi, Annalisa Bodina, Maria Gramegna, Silvana Castaldi, Danilo Cereda, Daniela De Angelis, Covid-19 Lombardy Working Group

**Affiliations:** 1 Department of Biomedical Sciences for Health, Postgraduate School of Public Health, Via Pascal 36, University of Milan, Milan, Italy; 2 MRC Biostatistics Unit, University of Cambridge, East Forvie Building, Robinson way, Cambridge, UK; 3 Department of Statistics, University of Warwick, Mathematical Sciences Building, Academic Loop Road, Warwick, UK; 4 Fondazione IRCCS Ca’ Granda Ospedale Maggiore Policlinico, University of Milan, Via Francesco Sforza, 28 Milan, Italy; 5 Welfare General Directorate, Regione Lombardia, Piazza Città di Lombardia 1, Milan, Italy

**Correction to: BMC Public Health (2021) 21:1612.**

**http://orcid.org/10.1186/s12889-021-11669-w**

It was highlighted that in the original article [1] the authors given and family names were interchanged. This Correction article shows the correct authorship. The original article has been updated.
